# Detection of ethyl carbamate in liquors using surface-enhanced Raman spectroscopy

**DOI:** 10.1098/rsos.181539

**Published:** 2018-12-12

**Authors:** Haiyan Qi, Huacai Chen, Yan Wang, Li Jiang

**Affiliations:** College of Optical and Electronic Technology, China Jiliang University, 310018 Hangzhou, China

**Keywords:** surface-enhanced Raman scattering (SERS), ethyl carbamate, flower-shaped silver nanostructure substrate, silver nanocubes, linear regression model

## Abstract

Ethyl carbamate (EC), a potential carcinogen, can be formed during the fermentation and storage of alcoholic beverages. In this work, quantitative detection of EC in alcoholic beverages by using surface-enhanced Raman spectroscopy (SERS) is reported. Flower-shaped silver nanostructure substrates and silver nanocube substrates were prepared and employed as SERS platform. Flower-like silver substrates had better Raman enhancement effect on EC and were selected for further EC detection. In EC SERS spectra based on flower-shaped silver substrates, the strongest and reproducible characteristic band at 857 cm^−1^ was chosen for establishing a linear regression model in the concentrations ranging from 10^−5^ to 10^−9^ M, which effectively extended the application scope of the quantitative model for determination EC. Furthermore, a real alcoholic beverage was tested to verify the feasibility and reliability of the method.

## 1. Introduction

Ethyl carbamate (EC, urethane), a by-product of fermentation and storage, is widely contained in most fermented food and alcoholic beverages [1]. The carcinogenic and mutagenic effect of EC to animals was detected in the 1940s [2]. In March 2007, EC was classified as a group 2A carcinogen (probably carcinogenic to humans) by the World Health Organization's International Agency for Research on Cancer [3]. The researches based on animal experiments have proved that EC can lead to the incidence of cancer in lung, blood vessels and liver, which means that EC is a recognized multi-site carcinogenic compound [4,5]. For the prevention and reduction of EC contamination, many countries including Canada, the United States, France, Japan, etc. have regulated legal limit levels for EC in alcoholic beverages ranging from 15 to 1000 µg l^−1^ [4,6–8]. Hence, many researches on quantitative detection of EC in alcoholic beverages and some fermented food have been done.

Several reliable methods of EC detection, including high-performance liquid chromatography combined with mass spectrometry detection [9], or fluorescence detection [10–12], gas chromatography (GC) equipped with packed columns and capillary columns [13], GC–tandem mass spectrometry [14,15], multidimensional GC [16], etc., have been developed. These methods show high sensitivity and reproducibility even though EC is at low concentration. However, extensive sample procedures (e.g. homogenization, extraction and cleanup) are often required, which is time-consuming and of relatively high cost. Therefore, a more rapid and sensitive method for quantification of EC is urgently required.

Surface-enhanced Raman scattering (SERS) is a promising technology with high sensitivity [17], convenient detection [18] and rapidity [19], which has been widely applied in environmental monitoring [20], chemical analysis [21,22], food security [23] and other fields [24,25]. Within the last few years, SERS has been initially applied by Yang to quantitative detection of EC in alcoholic beverages. In Yang's research, silver-coated gold nanoparticle colloids were used as SERS amplifiers, and the linear relationship between the SERS intensity and the EC concentration was found in the range from 1 × 10^−9^ to 1 × 10^−7^ M (8.9 µg l^−1^) [1]. Compared to the maximum limit level for largely consumed alcoholic beverages in several countries mentioned above (15–1000 µg l^−1^), the linear range is relatively narrow and the linear model could not be suitable for quantitative detection of EC in alcoholic beverages. Li applied Ag nanostar substrates for detection of EC in wine. The Raman intensity was increased linearly with an increase of EC concentration range from 5 × 10^−9^ M to 1 × 10^−4^ M and the detection limit was 1.37 × 10^−9^ M. However, the synthesis of Ag nanostars required stirring for at least 48 h, which was very time consuming [26]. It is necessary to develop a rapid and facile synthesis of nanostructure substrates for EC detection.

In this work, SERS was used for qualitative and quantitative detection of EC in different concentrations. Flower-shaped silver nanostructures and silver nanocubes were prepared as platforms for acquiring EC SERS spectra. Due to the abundant anisotropic protrusions, flower-like silver substrates can provide many more ‘hot spots’ than a regular structure. It was found that the enhancement of the flower-like silver substrates was better. The Raman shifts and the assignment of characteristic bands in the EC SERS spectra were summarized. The performance of flower-shaped silver substrates towards EC and their application in real samples was investigated, which revealed that the method could be used for qualitative and quantitative detection of EC in real alcoholic beverages effectively.

## 2. Material and methods

### 2.1. Chemicals and materials

Silver nitrate (AgNO_3_, 99.9%), ascorbic acid (ASA, greater than 99.0%), polyvinylpyrrolidone (PVP, K30, greater than 95% purity), EC (greater than 99.9%), rhodamine 6G (R6G) and sodium sulfide (Na_2_S) were purchased from Aladdin Reagent Co. Ltd (Shanghai, China). Ethylene glycol (EG) and acetone were purchased from Zhejiang Hannuo Chemical Reagent Company. Ultrapure water (greater than 18.3 MΩ) was used to prepare all the aqueous solutions. The liquor used in this research was purchased from a local supermarket.

### 2.2. Fabrication of flower-shaped silver nanostructures and silver nanocubes

Flower-shaped silver nanostructures: First, 0.2 ml of AgNO_3_ solution (1 M) and 2 ml of PVP solution (1%) were mixed successively with 10 ml of ultrapure water in a clean beaker. After mixing thoroughly, 1 ml of ASA solution (0.1 M) was quickly added into the above mixed solution, followed by stirring the solution persistently for 10 min. Next, the reaction product was separated from the solution by centrifugation at 5000 r.p.m. for 10 min and washed with ultrapure water. Then, flower-shaped silver nanostructure solution in 10 ml of ultrapure water was obtained [22].

Silver nanocubes: The procedures were according to the literature [27]. First, 10 ml of EG solution of Na_2_S (50 µM) was vigorously stirred after the addition of PVP (0.15 M). This mixed solution was injected into 10 ml of EG solution of AgNO_3_ (0.1 M) under magnetic stirring. The injection time was controlled in one minute. Next, the mixture was transferred into a 50 ml hydrothermal reactor and kept at 135°C for 1.5 h. When the reaction was finished, the reactor was naturally cooled to room temperature. Then, the products were centrifuged at 15 000 r.p.m. for 20 min and washed with acetone and ultrapure water, and dispersed in 10 ml of ultrapure water.

In comparison with colloidal nanoparticles, one advantage of solid substrates is that they allow much more flexibility [28]. Therefore, flower-shaped silver nanostructure solution (2 ml) and silver nanocube solution (2 ml) were each pipetted on silicon wafers (0.5 cm × 0.5 cm), and were dried in a vacuum oven at 60°C for 6 h. Flower-shaped silver substrates and silver nanocubes substrates were obtained.

### 2.3. Characterization

The flower-like silver substrates and silver nanocubes were characterized by field emission scanning electron microscopy (SEM; Hitachi SU8010). UV–visible absorption spectra of the synthesized silver nanostructures were recorded with a UV–visible spectrophotometer (TU-1901, Purkinje, China). The Raman spectra of the probe molecules and EC were measured with a confocal microscope/Raman spectrometer system (Horiba, LabRAM HR).

### 2.4. Surface-enhanced Raman scattering test

SERS test of R6G: The prepared substrates were each immersed in an aqueous solution of R6G ranging from 10^−5^ to 10^−9^ M, and dried at 60°C for 6 h in a vacuum oven. SERS spectra were acquired by a confocal microscope/Raman spectrometer system (Horiba, LabRAM HR) with 785 nm and 532 nm excitation wavelengths, with 25 mW power at the sample surface. The entire spectral window (from 300 to 2000 cm^−1^) was recorded. All the spectra were obtained under 50× objective with 5 s acquisition and single accumulation.

SERS test of EC: The prepared substrates were each immersed in EC aqueous solution ranging from 10^−5^ to 10^−9^ M, and dried at 60°C for 6 h in a vacuum oven. The measurement was carried out using the same procedure as described for R6G.

SERS test of EC in liquor: A certain amount of EC powder was dissolved in the liquor to make EC alcoholic solution for SERS measurement. Flower-shaped silver nanostructure substrate was immersed in the EC alcoholic solution, and taken out and dried under natural conditions. In order to reduce the experimental error, the measurement parameter setting is consistent with the measurement parameters of the EC aqueous solutions.

For flower-shaped silver substrate, a 785 nm excitation wavelength was an optimum wavelength. For silver nanocubes, a 532 nm excitation wavelength was an optimum wavelength. Except the excitation wavelength, other experimental parameters were consistent in all SERS tests. In addition, all SERS spectra were recorded by the confocal microscope/Raman spectrometer system (Horiba, LabRAM HR), and spectral data were background-subtracted by the spectral acquisition software (LabSpec 6.0 from Horiba Jobin Yvon) for the instrument we used.

## 3. Results and discussion

### 3.1. Substrates

**Figure 1.**
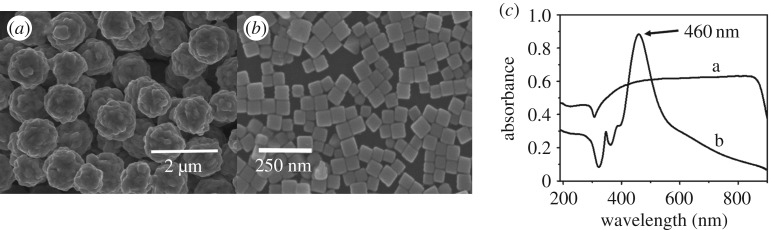
Characterization of the prepared substrates. (*a*) SEM image of flower-shaped silver nanostructures, (*b*) SEM image of silver nanocubes, (*c*) UV–visible spectra (a, flower-shaped silver nanostructures; b, silver nanocubes).

The prepared substrates were characterized by SEM. Flower-shaped silver nanostructures with diameters of 500 nm were well-distributed (figure 1*a*). For silver nanocubes, the SEM image is shown in figure 1*b*; the size of silver nanocubes was 50–60 nm. As illustrated in figure 1*c*, the UV–visible absorption spectra were measured in the range of 190–900 nm. There was a broad local surface plasma resonance region of flower-shaped silver nanostructures, and silver nanocubes exhibited an obvious absorption peak at 460 nm, which was consistent with Sosa's previous report [28].

**Figure 2.**
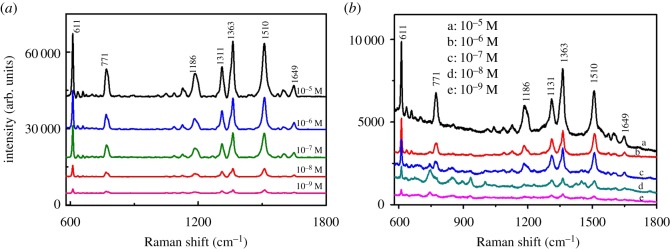
SERS spectra of R6G adsorbed on two different substrates: (*a*) flower-shaped silver substrates, (*b*) silver nanocube substrates.

For the purpose of evaluating the effectiveness of these prepared substrates, R6G was chosen as a nonresonant probe molecule owing to the well-established characteristic bands. Figure 2 exhibits the SERS spectra of R6G adsorbed on two different substrates. The Raman bands were clearly obtained and corresponded well with the previous reports on R6G [29]. The assignments for characteristic bands of R6G are shown in electronic supplementary material, table S1 [30]. The detection limit of R6G adsorbed on the two different silver substrates was the same (10^−9^ M), which revealed that the prepared substrates had good performance in enhancing the SERS signal of R6G. Both of them could be employed as SERS substrates for EC detection (figure 2).

**Figure 3.**
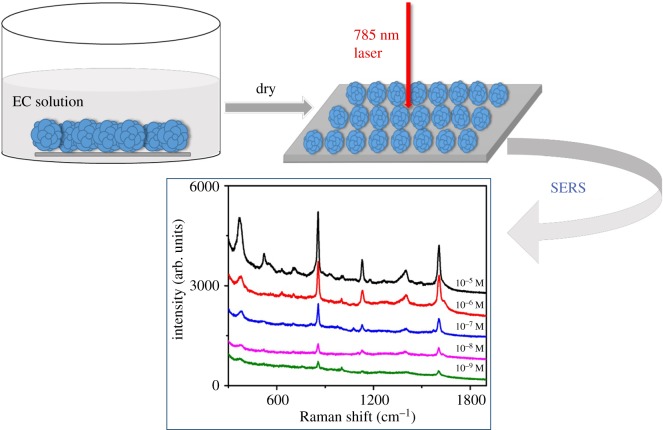
Schematic illustration of flower-shaped silver for SERS test of EC solution.

### 3.2. Qualitative and quantitative detection of ethyl carbamate

**Figure 4.**
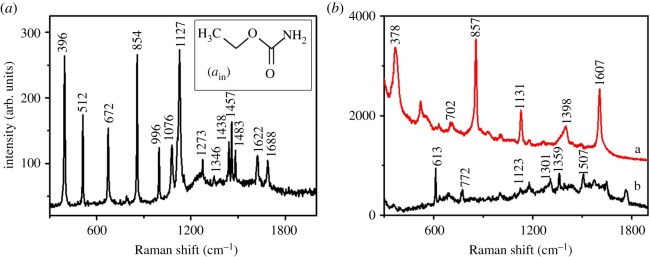
(*a*) The normal Raman spectrum of EC powder; (*a*_in_) the molecular structure of EC. (*b*) SERS spectra of EC solution (10^−5^ M) adsorbed on different substrates (a, flower-shape silver substrate; b, silver nanocube substrate).

Figure 4*b* shows the SERS spectra of EC solution (10^−5^ M) deposited on flower-shaped silver substrate and silver nanocubes. It is obvious that SERS signal of EC on the surface of flower-shaped silver substrate was stronger and easier to distinguish. The result could be attributed to the formation mechanism of flower-shaped silver nanostructure. The formation process was divided into two steps: Ag^+^ was firstly reduced to Ag^0^ monomer by the action of ASA, which also included pre-nucleation stage before cluster formation. When saturation state was reached, Ag^0^ monomer agglomerated and formed clusters. Then, the small clusters may grow bigger. Variation of free energy of crystal surface happened by the addition of PVP, which influenced the growth rate of crystal nuclei and resulted in the anisotropic growth. Flower-shaped silver nanoparticles finally formed and had better performance in enhancing SERS signals due to the numerous anisotropic protrusions as the sources of ‘hot spots’ on the surface [22]. Due to these numerous ‘hot spots’, the SERS signals of EC adsorbed on flower-shaped silver substrates could be effectively enhanced. Before being selected for further EC detection, the uniformity of flower-shaped silver substrates was also evaluated. The SERS spectra of R6G were randomly measured from 25 different areas of flower-shaped substrates, and the measurement parameter was consistent with the parameter set for R6G ranging from 10^−5^ to 10^−9^ M. The stability of R6G (10^−6^ M) band at 611 cm^−1^ based on flower-shaped silver substrates is shown in electronic supplementary material, figure S1a, and the stability of R6G (10^−6^ M) band at 1510 cm^−1^ based on flower-shaped silver substrates is shown in electronic supplementary material, figure S1b. The relative standard deviation of the intensity of R6G bands at 611 cm^−1^ and 1510 cm^−1^ was 8.16% and 7.74%, respectively, which demonstrated that these prepared flower-shaped silver substrates were highly uniform. Therefore, flower-shaped silver substrates were selected for further EC detection.

As is well known, the chemical bonds in a molecule have a corresponding vibration frequency, and the vibration frequency is related to the Raman scattering property. In other words, analysis of scattering bands can provide information about molecules and chemical bonds to characterize structure. EC (C_3_H_7_NO_2_) appears neutral in aqueous solution, and its molecular structure is shown in figure 4*a*_in_. Figure 4*a* illustrates the Raman spectrum of EC powder. According to the spectra of EC deposited on flower-shaped silver substrates, the bands at 378 (δ(OCC) + ω(CH)), 857 (ρ(NH_2_) + ρ(CH_3_)), 1311 (δ(NH_2_ + CH_3_)), 1399 (υs(CC) + υ(CH_3_)) and 1607 (β(NH_2_)) cm^−1^ were consistent with bands in normal spectrum (figure 4*a*). Discrepancies were also easily found in these two spectra. The bands at 378 and 672 cm^−1^ in SERS spectra, which were very intense in normal Raman spectrum, had been weakened. The bands at 857, 1131, 1399 and 1607 cm^−1^ were enhanced effectively in SERS spectra. As we found, the Raman enhancement effect on the bands at 857 and 1607 cm^−1^ was relatively more evident, which were assigned to the ρ(NH_2_) + ρ(CH_3_) rocking and the β(NH_2_) in-plane deformation respectively. In addition, small shifts in the SERS spectra were found in comparison with the Raman spectrum, which might be owing to the interaction of EC with flower-shaped silver nanostructure. All these bands could characterize the Raman scattering bands and be useful to tracing EC analysis.

**Figure 5.**
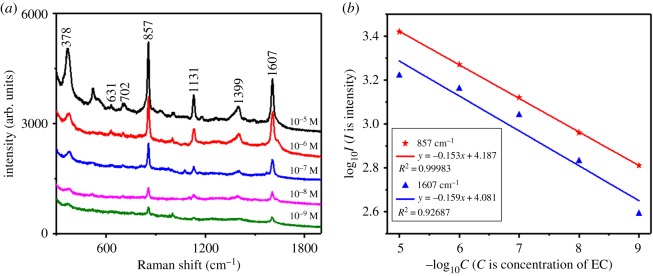
(*a*) SERS spectra of EC adsorbed on flower-shaped silver substrates in different concentrations (10^−5^ to 10^−9^ M). (*b*) The linear regression curves of EC adsorbed on flower-shaped silver substrates and the linear regression equations.

The SERS spectra of EC solution deposited on flower-shaped silver substrates at different concentrations ranging from 10^−5^ to 10^−9^ M were recorded (figure 5*a*). The characteristic bands at 378, 857, 1131, 1399 and 1607 cm^−1^ were clearly observed. Their assignments are shown in electronic supplementary material, table S2 [31]. With the decrease of concentrations, the SERS intensity of EC solution decreased correspondingly. The SERS signal still can be observed when the EC concentration dropped to 10^−9^ M, demonstrating that the prepared flower-shaped silver substrates had high sensitivity to EC and performed well in enhancing EC SERS signals. Meanwhile, the correlationship between the SERS intensity and EC concentration was explored. Based on the intensity of two characteristic bands (857 and 1607 cm^−1^), the regression models between the SERS intensity and EC concentration were established respectively. Figure 5*b* exhibits the linear regression curves of EC adsorbed on flower-shaped silver substrates and the linear regression equations. In these equations, *y* axis denotes log_10_*I* (*I* is intensity), and *x* axis represents log_10_*C* (*C* is EC concentration in the unit of M). The correlation coefficient (*R*^2^) is 0.99 and 0.93, respectively.

### 3.3. Determination of ethyl carbamate in liquor sample

**Figure 6.**
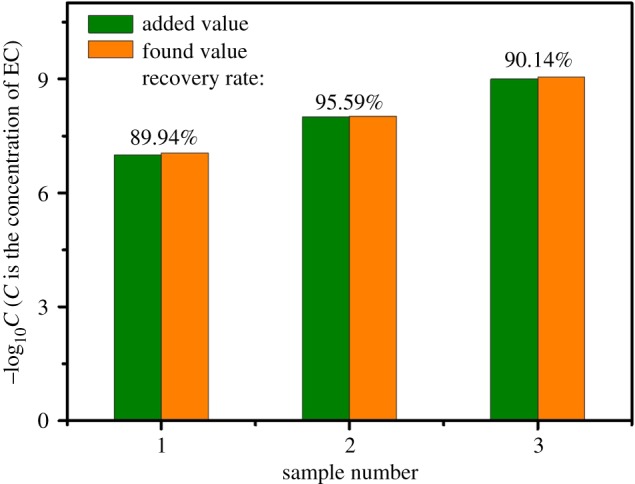
Results of EC detection based on flower-shaped silver substrates in real samples.

To investigate the practicality of the developed method, the flower-shaped sliver substrates were applied to the measurement of EC in real samples. The liquor sample was first analysed by the SERS measurement to ensure that there was no EC. Then, the liquor was spiked with three different concentrations of EC for SERS measurement. As shown in electronic supplementary material, figure S2, the characteristic band of EC at 857 cm^−1^ was clearly observed. Due to the higher *R*^2^, the linear regression model based on 857 cm^−1^ was used for calculating predicted value of the EC concentration in real samples. Figure 6 illustrates the results of error analysis between actual values and calculated values of EC, where *y* axis represents the concentration of EC and *x* axis is the identifier of the samples in different concentrations. The recovery rates of three concentrations of EC in the liquor were 89.94%, 95.59% and 90.14%, respectively, revealing that flower-shaped silver substrates had high accuracy for determination of EC in real samples.

## 4. Conclusion

In summary, SERS-based detection using flower-shaped silver nanostructure substrate provides a sensitive platform for sensing EC in water. Due to the numerous anisotropic protrusions as the sources of ‘hot spots’ on the surface of flower-shaped nanostructures, the substrate exhibited high performance in enhancing Raman signal and had good uniformity. The fabrication of flower-shaped silver substrates was simple and convenient, which was conducted in aqueous medium without heavy and organic wastes. The proposed method showed good analytical performance for EC with a wide linear range (10**^−^**^9^ to 10**^−^**^5^ M) for quantitative detection of EC. The detection limit of EC aqueous solution was 10**^−^**^9^ M (0.89 × 10**^−^**^11^ g l^−1^). There was a linear relationship between the SERS intensity and concentrations, and the correlation coefficient was 0.99. Moreover, flower-shaped silver substrates displayed good recovery rates for detection of EC in liquor samples, which is potentially a powerful tool for quantitative analysis of EC in further alcoholic beverages and other foodstuffs.

## Supplementary Material

Electronic supplementary material

## Supplementary Material

Fig.4b SERS spectra of EC adsorbed on different substrates.opj

## Supplementary Material

Fig.5 SERS specstra of EC and linear curve.opj

## Supplementary Material

Fig6 detection in real sample.opj

## Data accessibility

The datasets supporting this article have been uploaded as part of the electronic supplementary material.
