# Supplementary material for: Detection of ethyl carbamate in liquors using surface-enhanced Raman spectroscopy
Source: R Soc Open Sci. 2018 Dec 12;5(12):181539. doi: 10.1098/rsos.181539 (PMC6304119; doi:10.1098/rsos.181539)
Supplement: Electronic supplementary material [file rsos181539supp1.docx]

**Detection of ethyl carbamate in liquors using Surface-enhanced Raman spectroscopy**

Haiyan Qi, Huacai Chen*, Yan Wang, Li Jiang.

College of Optical and Electronic Technology, China Jiliang University, 310018,

Hangzhou, China.

Table S1 The assignment of the R6G main characteristic bands[1].

| SERS spectra(cm^-1^) | Vibrational assignment |
| --- | --- |
| 611 | δ(C-C-C) |
| 771 | δ(C-C-C) |
| 1131 | υ(C-H) |
| 1186 | δ(C-H) |
| 1363 | υ(C-C) |
| 1510 | υ(C-C) |
| 1649 | υ(C-C) |

Table S2 The assignment of the EC main characteristic bands of Raman spectrum and SERS spectra based on the flower-shaped silver substrates.[1]

| Raman spectrum(cm^-1^) | SERS spectra(cm^-1^) | Vibrational assignment |
| --- | --- | --- |
| 396 | 378 | δ(OCC) +ω(CH) |
| 854 | 857 | ρ(NH_2_) + ρ(CH_3_) |
| 1127 | 1131 | δ(NH_2_+CH_3_) |
| 1460 | 1399 | υs(CC) + υ(CH_3_) |
| 1690 | 1607 | β(NH_2_) |

Table S3 The assignment of EC main characteristic bands of SERS spectra based on silver nanocubes substrates. 

| Raman spectrum(cm^-1^) | SERS spectra(cm^-1^) | Vibrational assignment |
| --- | --- | --- |
| 672 | 613 | δ(C-C-C) |
| 854 | 772 | ρ(NH_2_) + ρ(CH_3_) |
| 1127 | 1123 | δ(NH_2_+CH_3_) |
| 1273 | 1301 | ρ(CH_3_+CH_2_) |
| 1346 | 1359 | ω(CH_2_) +δ(CNH) |
| 1483 | 1507 | δ(C-H) |

SERS, surface-enhanced Raman scattering; υ, stretching; δ, bending; ρ, rocking; ω, wagging; β, strong in-plane deformation; s, symmetric;


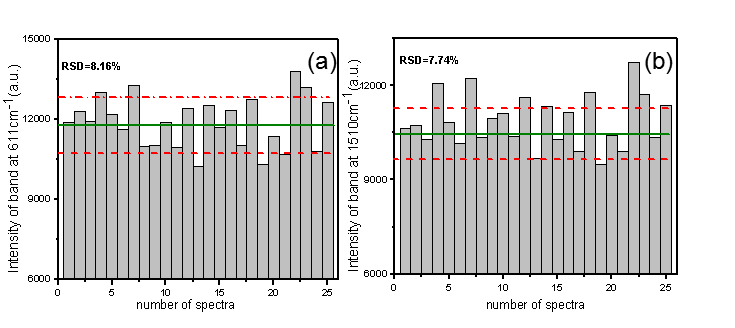


Fig. S1 The stability of R6G (10^-6^M) adsorbed on flower-shaped silver substrates (a:band at 611 cm^-1^, b:band at 1510 cm^-1^).


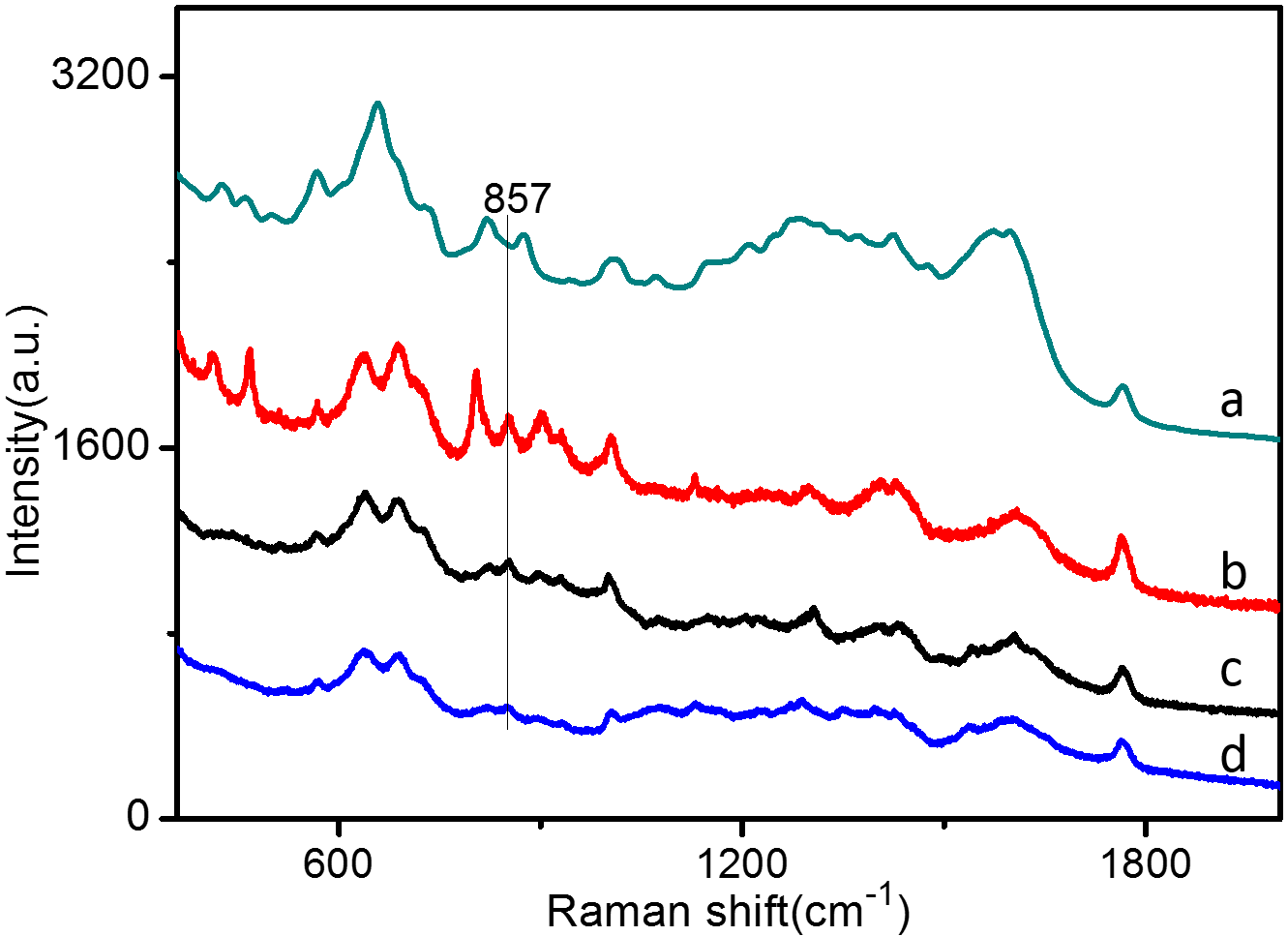


Fig. S2 The SERS spectra of the white spirit sample spiked with different concentrations of EC adsorbed on flower-shaped silver substrates (a: 0 M; b: 10^-7^ M; c: 10^-8^ M; d: 10^-9^ M).

**References:**

1. Yang, D.; Mircescu, N. E.; Zhou, H.; Leopold, N.; F, V. C. X.; Oltean, M.; Ying, Y.; Haisch, C., DFT study and quantitative detection by surface-enhanced Raman scattering (SERS) of ethyl carbamate †. Journal of Raman Spectroscopy 2013, 44, (11), 1491–1496.

2 Zhai W L, Li D W, Qu L L, et al. Multiple depositions of Ag nanoparticles on chemically modified agarose films for surface-enhanced Raman spectroscopy[J]. Nanoscale, 2011, 4(1):137-142.
